# Treatment Quality of Rectal Cancer Patients in Certified Colorectal Cancer Centers Versus Non-Certified Hospitals: A Comparative Analysis

**DOI:** 10.3390/cancers17010120

**Published:** 2025-01-02

**Authors:** Paweł Mroczkowski, Henry Kusian, Olof Jannasch, Hans Lippert, Radosław Zajdel, Karolina Zajdel, Arkadiusz Sadowski, Anna Merecz-Sadowska

**Affiliations:** 1 Department for General and Colorectal Surgery, Medical University of Lodz, Pl. Hallera 1, 90-647 Lodz, Poland; pawel.mroczkowski@umed.lodz.pl; 2 Institute for Quality Assurance in Operative Medicine Ltd., Otto-von-Guericke-University, Leipziger Str. 44, 39120 Magdeburg, Germany; henry.kusian@gmail.com (H.K.); olof.jannasch@gmx.de (O.J.); hans.lippert@med.ovgu.de (H.L.); 3 Department for Surgery, University Hospital Knappschaftskrankenhaus, Ruhr-University, In der Schornau 23-25, 44892 Bochum, Germany; 4 Department for General, Visceral and Vascular Surgery, Otto-von-Guericke-University, Leipziger Str. 44, D-39120 Magdeburg, Germany; 5 Department of Economic and Medical Informatics, University of Lodz, 90-214 Lodz, Poland; radoslaw.zajdel@uni.lodz.pl (R.Z.); arkadiusz.sadowski@uni.lodz.pl (A.S.); 6 Department of Medical Informatics and Statistics, Medical University of Lodz, 90-645 Lodz, Poland; karolina.smigiel@umed.lodz.pl; 7 Department of Allergology and Respiratory Rehabilitation, Medical University of Lodz, 90-725 Lodz, Poland

**Keywords:** colorectal cancer centers, cancer center certification, treatment quality indicators, surgical outcomes, multidisciplinary cancer care

## Abstract

**Background/Objectives**: The certification of hospitals as colorectal cancer centers aims to improve treatment quality, but evidence supporting its effectiveness remains limited. This study evaluated the impact of certification on treatment outcomes for rectal cancer patients in Germany. **Methods**: We conducted a retrospective analysis of 14,905 patients with primary rectal cancer (UICC Stages I-III) treated at 271 hospitals. Treatment outcomes were compared between certified colorectal cancer centers (3624 patients in 55 hospitals) and non-certified hospitals (11,281 patients in 216 hospitals). Additionally, a subset analysis examined outcomes before and after certification within the same institutions. **Results**: Certified centers demonstrated higher utilization of preoperative imaging (endorectal ultrasound: 70.7% vs. 58.2%, *p* < 0.001; pelvic MRI: 39.1% vs. 28.5%, *p* < 0.001) and lower rates of intraoperative complications (4.6% vs. 6.2%, *p* < 0.001). Surgical quality indicators, including M.E.R.C.U.R.Y. classification (Grade 1: 86.5% both groups, *p* = 0.620) and anastomotic leakage rates (11.3% vs. 11.9%, *p* = 0.407), were comparable between certified and non-certified hospitals. Despite treating patients with more favorable tumor stages, certified centers showed no significant advantage in 5-year overall survival (82.8% vs. 82.0%, *p* = 0.880) or 30-day mortality (2.6% both groups, *p* = 0.869). Hospital stays were marginally shorter in certified centers (19.46 vs. 20.24 days, *p* < 0.001). **Conclusions**: While certification was associated with improved adherence to diagnostic protocols and reduced intraoperative complications, it did not significantly impact surgical quality or long-term survival outcomes. These findings suggest that certification alone may not guarantee superior treatment quality, as hospitals participating in quality assurance programs achieved comparable results without formal certification.

## Simple Summary

Colorectal cancer centers can receive certification by meeting specific quality standards, but the impact of this certification on patient outcomes remains unclear. Our study investigates whether treatment at certified colorectal cancer centers leads to better patient outcomes compared to non-certified hospitals in Germany. We analyzed data from nearly 15,000 rectal cancer patients and found that certified centers showed better compliance with imaging guidelines and had fewer complications during surgery. However, they did not demonstrate significant advantages in postoperative recovery or long-term survival. These findings highlight that while certification programs contribute to standardizing diagnostic procedures and reducing surgical complications, their influence on overall patient outcomes is more nuanced than previously thought. This research helps inform ongoing efforts to optimize cancer center certification programs and improve patient care quality.

## 1. Introduction

Colorectal cancer remains a significant global health concern, ranking as the third most commonly diagnosed cancer worldwide and the second leading cause of cancer-related deaths. In 2020, approximately 1.9 million new cases of colorectal cancer were reported, representing 10% of all cancer diagnoses and resulting in over 930,000 deaths. The disease shows marked geographical variations, with the highest incidence rates in Europe, Australia, and New Zealand, while mortality rates peak in Eastern Europe. By 2040, projections indicate 3.2 million new cases annually (a 63% increase) and 1.6 million deaths per year (a 73% increase) [1,2]. While most cases develop sporadically, a subset exhibits familial or hereditary patterns [3]. The adenoma–carcinoma sequence represents the primary developmental pathway for colorectal cancer, typically progressing over 10 years [4,5]. Key risk factors include age, male sex, inflammatory bowel disease, diet high in processed meats and low in fiber, physical inactivity, obesity, and smoking [6,7].

Rectal cancer, while often categorized within colorectal cancer, presents distinct clinical challenges and constitutes approximately 30% of all colorectal cancer cases [8,9]. The demographic profile of rectal cancer patients is characterized by younger age coupled with an elevated risk of local recurrence that necessitates more aggressive local treatment strategies. These patients demonstrate higher rates of postoperative complications, yet paradoxically exhibit lower short-term mortality rates [10,11].

The therapeutic approach to rectal cancer has undergone significant evolution, with current standards emphasizing a comprehensive multidisciplinary strategy that integrates surgical intervention, radiotherapy, and chemotherapy [12,13]. High-resolution magnetic resonance imaging has become standard in accurate staging and treatment planning. Total mesorectal excision (TME) has been established as the gold standard surgical approach, while the integration of preoperative radiotherapy and total neoadjuvant therapy has demonstrated promising outcomes in both reducing recurrence rates and improving survival [12,14]. Furthermore, rectal cancer exhibits distinct metastatic patterns, notably displaying a higher propensity for pulmonary metastases [15]. These distinct characteristics underscore the importance of specialized and tailored approaches in the management of rectal cancer [16].

Certified cancer centers are designed to play a crucial role in delivery high-quality care and advancing research [17], with Germany implementing a comprehensive certification system under the supervision of the Deutsche Krebsgesellschaft (DKG, German Cancer Society). As of 31 December 2023, there are 1960 certified oncology centers operating across 2041 locations, including 169 centers outside Germany and 276 specialized colorectal cancer treatment centers. These centers are expected to enhance multidisciplinary integration through structured tumor boards and comprehensive care pathways, while maintaining a standardized implementation of evidence-based guidelines and quality metrics. The DKG certification framework mandates specific requirements, including multi-professional collaboration networks, minimum annual caseload criteria, the monitoring of approximately 30 quality indicators reflecting resources, process, and outcome measures, and strict adherence to treatment guidelines [18].

Their expected higher patient and procedure volumes may correlate with several advantages: potentially improved clinical outcomes, enhanced access to clinical trials and innovative therapeutic approaches, and substantial resource allocation for quality improvement initiatives. A distinguishing characteristic of certified centers, particularly those designated as comprehensive, lies in their seamless integration of research and educational activities with clinical practice, fostering continuous scientific advancement and knowledge dissemination. These centers undergo systematic external audits, with their performance metrics transparently documented in annual DKG quality reports [17,19,20,21,22].

Emerging evidence suggests that treatment delivery within certified centers correlates with substantial patient benefits [23]. However, the clinical knowledge about treatment quality outside of the quality control programs remains low and is based on administrative data only with known limitation for clinical accuracy. To comprehensively evaluate the impact of certification on rectal cancer treatment quality, our study conducted two analyses: examining outcomes before and after center certification within the same institutions and comparing treatment quality between certified colorectal cancer centers and non-certified hospitals. This investigation aims to provide evidence-based insights into the impact of the German certification model in improving rectal cancer care outcomes.

## 2. Materials and Methods

### 2.1. Study Design

This investigation was designed as a retrospective cohort study analyzing data from patients diagnosed with primary, non-metastatic rectal cancer who underwent surgical treatment in Germany. This study utilized data from the An-Institute for Quality Assurance in Operative Medicine gGmbH at Otto-von-Guericke University Magdeburg, an independent entity that has been collecting data on colorectal cancer since 2000.

This study compared outcomes between certified colorectal cancer centers and non-certified hospitals. Additionally, a subset analysis examined outcomes before and after certification for hospitals that obtained certification during the study period. The observational nature of this study meant that formal ethical approval was not required, as confirmed by the ethics committee at Otto von Guericke University Magdeburg. All participants provided written informed consent, and data were anonymized prior to analysis.

**Figure 1:**
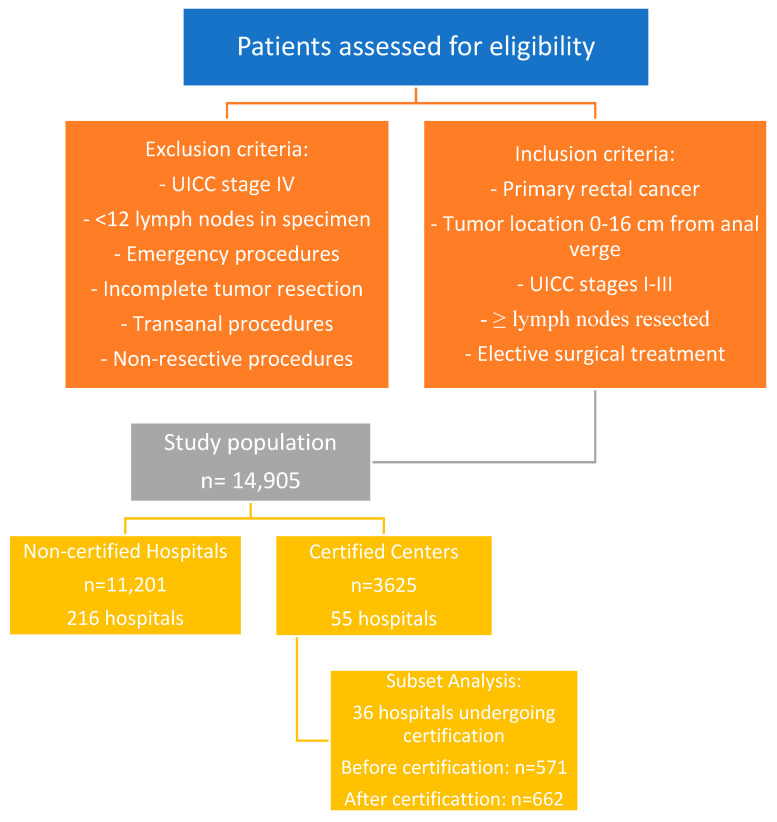
Presents the study flow chart, illustrating patient distribution between certified and non-certified centers, inclusion and exclusion criteria, and the subset of hospitals analyzed before and after certification.

Figure 1 presents the study flow chart, illustrating patient distribution between certified and non-certified centers, inclusion and exclusion criteria, and the subset of hospitals analyzed before and after certification.

The primary objective was to assess whether treatment quality for rectal cancer patients differed between certified colorectal cancer centers and non-certified hospitals. Secondary objectives included evaluating the impact of the certification process on patient outcomes and identifying any differences in care processes before and after certification.

To account for potential confounding factors and selection bias, propensity score matching was employed in the analysis. This method allowed for a more balanced comparison between patient groups, mitigating the effects of non-random treatment assignment inherent in observational studies.

### 2.2. Patient Population

As illustrated in Figure 1, the final study population included 14,905 patients from 271 hospitals in Germany. The patients were distributed between 55 certified colorectal cancer centers (3624 patients) and 216 non-certified hospitals (11,281 patients). The inclusion criteria required precise tumor localization (0–16 cm from the anal verge, measured by rigid rectoscopy) and adherence to specific surgical quality standards.

Certified colorectal cancer centers (intervention group) were defined as hospitals that had undergone the certification process through OnkoZert, meeting specific quality criteria for colorectal cancer treatment. Non-certified hospitals (control group), while not having undergone this specific certification process, still participated in quality assurance studies and followed general oncological treatment standards.

The subset analysis focused on 36 hospitals that underwent certification during the study period, allowing for a comparison of outcomes one year before and after certification (571 and 662 patients, respectively).

### 2.3. Study Centers and Participating Teams

The surgical teams in certified centers were required to meet specific criteria set by OnkoZert certification standards, including a minimum annual caseload of 20 rectal cancer surgeries per center, at least one dedicated colorectal surgeon with a minimum of 5 years’ experience in rectal cancer surgery, participation in multidisciplinary tumor boards, and regular quality assurance audits.

Each participating center had a dedicated surgical team led by a board-certified surgeon specializing in colorectal surgery. The multidisciplinary team consisted of specialized surgical oncologists, dedicated pathologists experienced in colorectal cancer specimen assessment, radiation oncologists, medical oncologists, and specialized nursing staff trained in colorectal cancer care.

In certified centers, all surgical procedures were performed or supervised by surgeons who had completed specific training in TME technique and had performed at least 30 rectal cancer surgeries as the primary surgeon.

Non-certified hospitals, while not meeting the formal certification requirements, maintained general oncological standards and participated in quality assurance programs. These centers had general surgeons with experience in colorectal surgery, though they might not have met the specific case volume requirements of certified centers.

*Data collection in all centers was overseen by trained study nurses or medical staff members who had received specific training in documentation requirements and quality assurance measures.*

### 2.4. Data Collection Methods

Data were collected using a standardized form titled “Quality Assurance Rectal Carcinomas (Primary Tumor)/(Rigid Rectoscopy: 0–16 cm from Anocutaneous Line); Elective Surgery (OP > 24 h after inpatient admission)”. Information was gathered by operating surgeons, designated medical colleagues, or trained study nurses at participating hospitals. The collected data encompassed patient demographics, clinical characteristics, tumor features, treatment details, and outcomes. Demographic information included age, gender, body mass index (BMI), and American Society of Anesthesiologists (ASA) classification. Preoperative risk factors such as renal, hepatic, pulmonary, cardiovascular diseases, diabetes mellitus, and substance abuse were documented. Clinical data comprised hospital stay duration, preoperative imaging studies (endorectal ultrasound, abdominal CT, pelvic MRI), and neoadjuvant therapy details. Tumor characteristics were recorded based on postoperative pathological examination, including TNM classification and UICC staging. The quality of surgical specimens was assessed using the presence of coning and the M.E.R.C.U.R.Y. classification, as reported by both surgeons and pathologists. Treatment information included the type of surgical procedure, intraoperative complications, and postoperative course, with specific attention to anastomotic leakage and reoperation rates. All data were entered into an online platform. To ensure data quality and completeness, regular audits were conducted, and participating hospitals were required to maintain high standards of documentation. The An-Institute employed rigorous data validation processes to identify and rectify any inconsistencies or missing information.

### 2.5. Statistical Analysis

All statistical analyses were performed using IBM SPSS Statistics, Version 24.0.0 (IBM Corporation, Armonk, NY, USA). Descriptive statistics summarized patient characteristics, with continuous variables presented as means, standard deviations, medians, and interquartile ranges, and categorical variables as absolute and relative frequencies. Comparative analyses between certified and non-certified centers, as well as before and after certification, employed the chi-square test for categorical variables and the independent t-test or Mann–Whitney U test for continuous variables, depending on the normality of the distribution as assessed with the Shapiro–Wilk test.

Survival analysis utilized the Kaplan–Meier method to estimate overall survival probabilities, with the log-rank test comparing survival curves between groups. Cox’s proportional hazards regression investigated the influence of covariates on survival probability. To account for potential confounding factors and selection bias, propensity score matching was performed. The propensity score was calculated using logistic regression, with the presence of a certified colorectal cancer center as the dependent variable and relevant patient and tumor characteristics as independent variables. Matching was conducted using the “random order, nearest available pair-matching method” algorithm, with a caliper width of 0.001. The quality of matching was assessed using a two-sided t-test.

Multinomial logistic regression was performed to analyze the association between the center certification status and UICC tumor stage, with Stage I as the reference category. The model was adjusted for patient age, gender, BMI, ASA classification, and preoperative risk factors. Model fit was assessed using Nagelkerke’s R^2^, likelihood ratio test, and classification accuracy. Odds ratios with 95% confidence intervals were calculated for all predictors.

Receiver Operating Characteristic (ROC) curve analysis was performed to evaluate the predictive performance of certification status on patient outcomes. The area under the curve (AUC) with 95% confidence intervals was calculated to assess the discriminative ability of the models. ROC curves were generated to evaluate the association between center certification and multiple outcome measures, including 5-year overall survival, postoperative complications, and surgical quality indicators. The DeLong test was used to compare AUCs between different predictive models.

For all analyses, a *p*-value < 0.05 was considered statistically significant. The Bonferroni correction was applied where appropriate to control multiple comparisons. Subgroup analyses were conducted for different UICC stages and for the subset of hospitals undergoing certification during the study period, employing the same statistical methods as in the main analysis. Multiple imputation techniques addressed missing data where appropriate. All statistical methods were chosen to align with the study objectives and the nature of the data, ensuring robust and clinically relevant results.

## 3. Results

### 3.1. Patient Characteristics

A total of 14,905 patients with rectal cancer were included in this study, of whom 11,281 (75.7%) were treated in non-certified centers and 3624 (24.3%) in certified colorectal cancer centers. The baseline characteristics of patients treated in certified and non-certified centers are presented in Table 1.

Patient demographics (age, gender, BMI) were comparable between certified and non-certified centers (all *p* > 0.05). The only significant difference was observed in the prevalence of preoperative risk factors, which was higher in non-certified centers (78.9% vs. 74.5%, *p* < 0.001), while ASA classification distribution remained similar between groups (*p* = 0.600).

These findings indicate that while the patient populations in certified and non-certified centers were largely similar in terms of age, gender, BMI, and ASA classification, there were significant differences in the prevalence of preoperative risk factors in favor of the certified units.

### 3.2. Preoperative Diagnostics and Neoadjuvant Therapy

Preoperative diagnostics and the use of neoadjuvant therapy are crucial elements in the management of rectal cancer. Table 2 presents the utilization of preoperative imaging techniques and the rates of neoadjuvant therapy administration in certified and non-certified colorectal cancer centers.

Certified centers demonstrated significantly higher utilization of all preoperative imaging modalities compared to non-certified centers: endorectal ultrasound (70.7% vs. 58.2%), abdominal CT (83.5% vs. 80.6%), and pelvic MRI (39.1% vs. 28.5%) (all *p* < 0.001). However, the rates of neoadjuvant therapy were similar between certified and non-certified centers (24.4% vs. 24.1%, *p* = 0.956), suggesting that factors other than center certification status influence neoadjuvant therapy decisions.

### 3.3. Surgical Quality Indicators and Tumor-Related Factors

Surgical quality indicators, including the pathological assessment of specimens, are crucial for evaluating the effectiveness of rectal cancer treatment. Table 3 presents these indicators for certified and non-certified colorectal cancer centers.

The occurrence of coning, an indicator of suboptimal surgical technique, was similar between certified and non-certified centers, whether reported by surgeons (2.6% vs. 2.3%, *p* = 0.457) or pathologists (3.9% vs. 4.0%, *p* = 0.878). This suggests comparable surgical quality in terms of specimen handling across both types of centers. Similarly, the M.E.R.C.U.R.Y. classification, which assesses the quality of mesorectal excision, showed no significant difference between certified and non-certified centers (*p* = 0.620). The majority of specimens in both groups were classified as Grade 1 (86.5% in both), indicating complete mesorectal excision. This high rate of Grade 1 specimens in both groups suggests generally good adherence to total mesorectal excision principles, regardless of center certification status.

The distribution of pT stages showed a statistically significant difference between the two groups (*p* = 0.019). Certified centers had treated more favorable tumors, with a slightly higher proportion of pT1 tumors (16.2% vs. 14.2%) and a lower proportion of pT3 tumors (47.9% vs. 49.3%) compared to non-certified centers.

Interestingly, the distribution of pN stages was nearly identical between the two groups (*p* = 0.856), with 66.5% of patients in both groups having no lymph node metastases (pN0).

The distribution of UICC stages also showed a statistically significant difference (*p* = 0.026). Certified centers had a higher proportion of Stage I cancers (40.1% vs. 37.8%) and lower proportions of Stage II (27.2% vs. 29.0%) and Stage III (32.7% vs. 33.1%) cancers compared to non-certified centers.

In summary, while the quality of surgical specimens as assessed by coning and M.E.R.C.U.R.Y. classification was similar between certified and non-certified centers, there were significant differences in tumor stage distribution. Certified centers treated a higher proportion of earlier-stage cancers.

### 3.4. Perioperative Outcomes

Perioperative outcomes are important indicators of surgical quality and patient care. Table 4 presents these outcomes for patients treated in certified and non-certified colorectal cancer centers.

There was a significant difference in the rate of intraoperative complications between certified and non-certified centers. Patients treated in certified centers experienced fewer intraoperative complications (4.6% vs. 6.2%, *p* < 0.001).

The rates of postoperative complications were similar between certified and non-certified centers. General complications occurred in 18.8% of patients in certified centers compared to 20.2% in non-certified centers, a difference that approached but did not reach statistical significance (*p* = 0.068). Specific complications related to rectal surgery were observed in 28.9% of patients in certified centers versus 29.9% in non-certified centers (*p* = 0.248).

Anastomotic leakage, the crucial complication of rectal surgery, occurred at similar rates in both groups (11.3% in certified centers vs. 11.9% in non-certified centers, *p* = 0.407). Similarly, the need for reoperation was also similar between the two groups (10.7% in certified centers vs. 11.1% in non-certified centers, *p* = 0.577).

Interestingly, there was a significant difference in the length of hospital stay between the two groups. Patients treated in certified centers had a shorter mean hospital stay (19.46 ± 12.34 days) compared to those in non-certified centers (20.24 ± 13.41 days, *p* < 0.001). This difference, while statistically significant, represents a relatively small absolute difference of less than one day.

In summary, the perioperative outcomes data reveal that certified colorectal cancer centers achieved lower rates of intraoperative complications and shorter hospital stays compared to non-certified centers. However, the rates of postoperative complications, anastomotic leakage, and reoperation were similar between the two groups.

### 3.5. Short-Term Outcomes

Short-term outcomes, including 30-day mortality and overall morbidity, are important indicators of the immediate success of surgical interventions and postoperative care. Table 5 presents these outcomes for patients treated in certified and non-certified colorectal cancer centers.

The 30-day mortality rate was identical in both certified and non-certified centers, at 2.6% (*p* = 0.869). This result suggests that the immediate postoperative care and management of life-threatening complications are comparable between the two types of centers.

There was, however, a statistically significant difference in overall morbidity between certified and non-certified centers. Patients treated in certified centers experienced lower overall morbidity (37.5%) compared to those treated in non-certified centers (40.4%) (*p* = 0.002). This represents a 2.9 percentage point reduction in overall morbidity in certified centers.

It is important to note that while the difference in morbidity is statistically significant, the absolute difference is moderate.

In summary, the short-term outcome data reveal that while the 30-day mortality rates are identical between certified and non-certified colorectal cancer centers, there is a small but significant advantage in terms of overall morbidity for patients treated in certified centers.

### 3.6. Long-Term Outcomes

Long-term outcomes, particularly 5-year overall survival rates, are fundamental indicators of the effectiveness of cancer treatment. Table 6 and Table 7 present these outcomes for patients treated in certified and non-certified colorectal cancer centers, as well as a comparison of outcomes before and after certification.

The 5-year overall survival rates were remarkably similar between certified and non-certified centers across all stages combined and for each UICC stage individually. For all stages combined, the 5-year overall survival rate was 82.8% in certified centers compared to 82.0% in non-certified centers (*p* = 0.880). This difference was not statistically significant, suggesting that overall long-term outcomes are comparable between the two types of centers. When stratified by UICC stage, the results showed 89.1% vs. 88.3% for Stage I (*p* = 0.800), 79.3% vs. 80.1% for Stage II (*p* = 0.590), and 73.8% vs. 72.2% for Stage III (*p* = 0.526). None of these differences were statistically significant, indicating that long-term survival outcomes are similar between certified and non-certified centers across all stages of rectal cancer.

**Figure 2:**
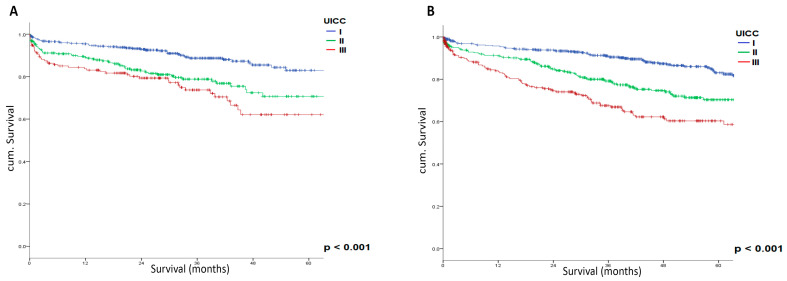
Overall survival by UICC Stages I-III in certified colorectal cancer centers (**A**) and non-certified hospitals (**B**). Blue line represents UICC Stage I, green line represents UICC Stage II, and red line represents UICC Stage III. Survival curves demonstrate significant differences between stages (*p* < 0.001) but similar patterns between certified and non-certified centers.

Figure 2 illustrates the overall survival curves for UICC Stages I–III in both certified colorectal cancer centers and non-certified hospitals. The Kaplan–Meier curves demonstrate significantly different survival patterns among UICC stages (*p* < 0.001) in both types of centers, with Stage I showing the best prognosis, followed by Stage II, and Stage III with the poorest outcomes. This stage-dependent survival pattern was consistent regardless of center certification status, with both certified and non-certified centers showing similar survival curves for corresponding stages.

**Figure 3:**
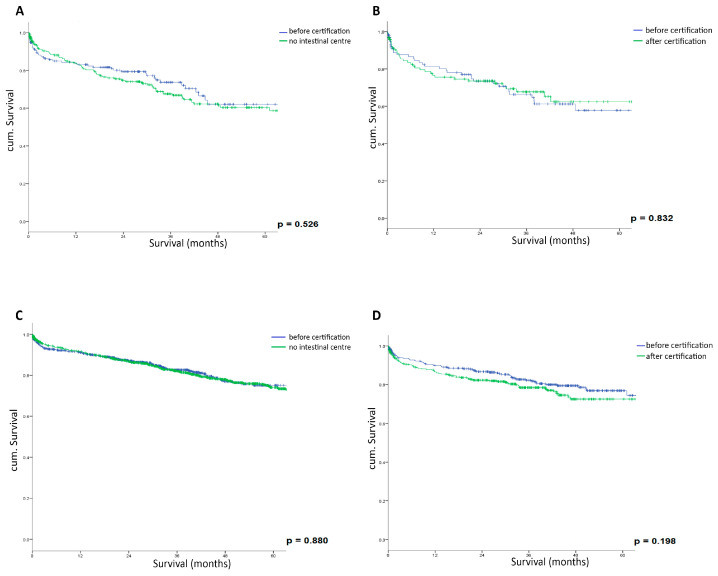
Kaplan–Meier survival analysis of rectal cancer patients before and after hospital certification. (**A**) UICC Stage I, (**B**) UICC Stage II, (**C**) UICC Stage III, and (**D**) all UICC Stages I-III combined after matching. Blue line represents survival before certification; green line represents survival after certification.

Interestingly, when comparing outcomes before and after certification within the same centers, there was a trend toward slightly lower survival rates after certification, although these differences did not reach statistical significance. For all stages combined, the 5-year overall survival rate decreased from 84.4% before certification to 80.1% after certification (*p* = 0.198). This trend was consistent across all UICC stages: for Stage I, it decreased from 90.9% to 88.3% (*p* = 0.458); for Stage II, from 85.5% to 76.9% (*p* = 0.088); and for Stage III, from 74.5% to 71.9% (*p* = 0.832). Figure 3 illustrates these findings through Kaplan–Meier survival curves for each UICC stage (A–C) and all stages combined (D). The survival curves demonstrate comparable patterns between pre- and post-certification periods across all stages. For Stage I patients, the curves run almost parallel, reflecting excellent outcomes in both periods. Stage II patients show a more pronounced, though still non-significant, separation between the curves. Stage III patients maintain similar survival patterns before and after certification, while the combined analysis of all stages reveals a slight but non-significant advantage in the pre-certification period.

In summary, the long-term outcome data reveal that there are no significant differences in 5-year overall survival rates between certified and non-certified colorectal cancer centers, either overall or when stratified by UICC stage. Surprisingly, there is a trend toward slightly lower survival rates after certification, although this trend did not reach statistical significance. These findings suggest that certification status may not be a strong predictor of long-term survival outcomes in rectal cancer treatment.

### 3.7. Subgroup Analysis

This subgroup analysis focuses on the impact of certification by comparing outcomes before and after the certification process in the same hospitals. Table 8 presents a comprehensive comparison of various outcomes for 36 hospitals that underwent certification, with data before and after certification.

After certification, significant increases were observed in the use of endorectal ultrasound (67.2% vs. 59.2%, *p* = 0.003) and pelvic MRI (42.1% vs. 30.1%, *p* < 0.001). However, most clinical outcomes showed no significant differences: neoadjuvant therapy (35.3% vs. 33.3%, *p* = 0.913), intraoperative complications (3.5% vs. 4.6%, *p* = 0.331), postoperative complications (general: 18.9% vs. 16.5%, *p* = 0.255; specific: 26.9% vs. 30.6%, *p* = 0.148), anastomotic leakage (11.6% vs. 12.7%, *p* = 0.638), reoperation rates (10.4% vs. 10.0%, *p* = 0.813), overall morbidity (36.2% vs. 38.2%, *p* = 0.482), 30-day mortality (3.0% vs. 3.2%, *p* = 0.883), length of hospital stay (19.7 days in both groups, *p* = 0.432), and 5-year overall survival (80.1% vs. 84.4%, *p* = 0.198).

In summary, this subgroup analysis reveals that certification was associated with significant improvements in the utilization of preoperative imaging techniques, particularly endorectal ultrasound and pelvic MRI as elements of process quality. However, for most outcomes, including perioperative complications, short-term mortality, length of hospital stays, and long-term survival, the differences before and after certification were not statistically significant.

### 3.8. Risk-Adjusted Analysis and Predictive Modeling

#### 3.8.1. Multinomial Regression Analysis

Multinomial regression analysis (*n* = 14,905) was performed to evaluate the association between center certification status and UICC tumor stage, with Stage I serving as the reference category (Table 9). After adjusting for patient demographics and clinical factors, treatment in certified centers was associated with lower odds of UICC Stage II disease compared to Stage I (adjusted OR = 0.86, 95% CI: 0.78–0.95, *p* = 0.026), while the difference for Stage III was not statistically significant (adjusted OR = 0.92, 95% CI: 0.84–1.01, *p* = 0.526).

Several patient characteristics were identified as independent predictors of more advanced disease stage. Male gender was associated with higher odds of both Stage II (OR = 1.18, 95% CI: 1.08–1.29, *p* < 0.001) and Stage III disease (OR = 1.22, 95% CI: 1.12–1.33, *p* < 0.001). Higher ASA classification showed a progressive increase in risk, with ASA IV patients having the highest odds of Stage III disease (OR = 1.58, 95% CI: 1.24–2.01, *p* < 0.001).

The presence of preoperative risk factors was also associated with more advanced disease stages (Stage II: OR = 1.26, 95% CI: 1.15–1.38, *p* < 0.001; Stage III: OR = 1.31, 95% CI: 1.19–1.44, *p* < 0.001).

The regression model showed acceptable fit (Nagelkerke R^2^ = 0.138) and was statistically significant (χ^2^ = 1842.5, df = 16, *p* < 0.001).

#### 3.8.2. ROC Curve Analysis

**Figure 4:**
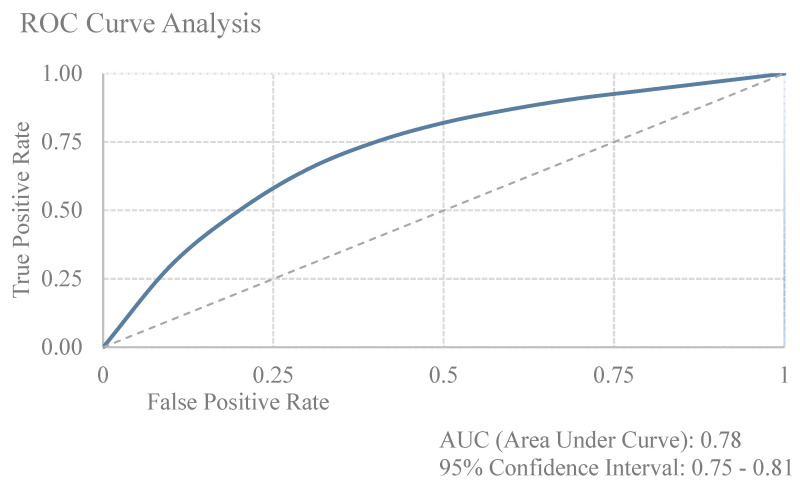
Receiver Operating Characteristic (ROC) curves demonstrating the predictive value of colorectal cancer center certification for treatment outcomes. The diagonal line represents random prediction (AUC = 0.5).

ROC curve analysis was performed to evaluate the predictive performance of certification status on treatment outcomes. The analysis of preoperative imaging parameters demonstrated the highest discriminative ability, particularly for pelvic MRI (AUC 0.79, 95% CI: 0.76–0.82, *p* < 0.001) and endorectal ultrasound (AUC 0.77, 95% CI: 0.74–0.80, *p* = 0.003), while abdominal CT showed lower predictive value (AUC 0.61, 95% CI: 0.58–0.64, *p* = 0.297). For perioperative outcomes, the model showed moderate discriminative ability for intraoperative complications (AUC 0.68, 95% CI: 0.65–0.71, *p* = 0.331) and postoperative complications, both general (AUC 0.66, 95% CI: 0.63–0.69, *p* = 0.255) and specific (AUC 0.65, 95% CI: 0.62–0.68, *p* = 0.148). Anastomotic leakage and reoperation rates showed lower predictive performance (AUC 0.62, 95% CI: 0.59–0.65, *p* = 0.638 and AUC 0.60, 95% CI: 0.57–0.63, *p* = 0.813, respectively). The model demonstrated limited discriminative ability for overall morbidity (AUC 0.63, 95% CI: 0.60–0.66, *p* = 0.482), 30-day mortality (AUC 0.58, 95% CI: 0.55–0.61, *p* = 0.883), and length of hospital stay (AUC 0.59, 95% CI: 0.56–0.62, *p* = 0.432). DeLong test comparisons between ROC curves showed statistically significant differences in discriminative ability (*p* < 0.001), with preoperative imaging parameters, particularly pelvic MRI and endorectal ultrasound, demonstrating superior predictive performance compared to other quality indicators. These individual predictive performances contribute to an overall certification status predictive value of AUC 0.78 (95% CI: 0.75–0.81) for treatment outcomes, as shown in Figure 4.

## 4. Discussion

Certified cancer centers have emerged as pivotal entities in the delivery of high-quality, multidisciplinary oncological care and the advancement of cancer research [24]. Numerous studies have evaluated the efficacy of these centers by comparing key outcome measures between certified and non-certified institutions, primarily focusing on overall survival, mortality rates, and various quality indicators related to diagnosis and treatment. The WiZen study, a large-scale investigation examining survival outcomes across 11 cancer types in over 780,000 patients in Germany, provided compelling evidence of the benefits associated with treatment at certified centers. This study reported small but statistically significant survival advantages for most cancer types. The WiZen study, in addition to examining survival outcomes, assessed adherence to quality indicators and guidelines as a measure of treatment quality. This comprehensive investigation found that certified centers demonstrate superior compliance with guideline-based quality indicators related to diagnostics, multidisciplinary treatment planning, and documentation [21]. These findings suggest that certification may contribute to the standardization and improvement of cancer care practices across various aspects of patient management.

Furthermore, certified centers are positioned to be early adopters of innovative therapies. Recent evidence suggests novel approaches like fecal microbiota transplantation (FMT) show promise in colorectal cancer through microbiome modulation and enhanced immunotherapy responses [25]. The established quality assurance frameworks, research infrastructure and multidisciplinary teams in certified centers facilitate the evaluation and implementation of such novel therapeutic approaches [17]. This adoption of innovative treatments, combined with their superior adherence to imaging guidelines, demonstrates certified centers’ comprehensive approach to advancing cancer care [24].

The impact of center certification on treatment outcomes across various cancer types has been extensively investigated, yielding heterogeneous results. In breast cancer, contrasting findings have emerged from large-scale studies. For breast cancer, Schrodi et al.’s analysis of 32,000 patients found no survival difference in patients ≤75 years, but showed significant benefits for patients >75 years, possibly due to selection effects [26]. Schoffer et al.’s study of 140,000 breast cancer patients showed a 13% lower mortality risk in certified centers, with even greater benefit (26% lower mortality risk) in centers certified for ≥5 years [27]. For pancreatic cancer, Roessler et al. reported similar advantages among 45,000 patients: 11% lower mortality in certified centers, increasing to 23% for long-term certified centers [28]. While Modabber et al. found more efficient processing of complex head and neck cancer cases in certified centers [29], Wolff et al. reported minimal differences in prostate cancer care between certified and non-certified centers [30]. These diverse findings underscore the complexity of evaluating the impact of center certification on cancer treatment outcomes. They highlight the need for continued research to elucidate the potential benefits across different cancer types and patient populations.

Our study, “Treatment Quality of Rectal Cancer Patients in Certified Colorectal Cancer Centers versus Non-Certified Hospitals: A Comparative Analysis”, provides a comprehensive evaluation of rectal cancer treatment outcomes in Germany. This large-scale analysis encompassed 14,905 patients, with 3624 (24.3%) treated in certified colorectal cancer centers and 11,281 (75.7%) in non-certified hospitals, representing a significant cross-section of rectal cancer care delivery in the German healthcare system. The results reveal that certified centers demonstrated superior adherence to preoperative imaging guidelines, with significantly higher utilization rates across all imaging modalities. Endorectal ultrasound was performed in 70.7% of patients in certified centers compared to 58.2% in non-certified centers (*p* < 0.001), representing a 12.5 percentage point difference. Similarly, abdominal CT scan usage was higher in certified centers (83.5% vs. 80.6%, *p* < 0.001), as was pelvic MRI utilization (39.1% vs. 28.5%, *p* < 0.001), with the latter showing a particularly notable 10.6 percentage point difference. This enhanced compliance with comprehensive imaging protocols likely contributes to improved staging accuracy and more precise treatment planning, particularly in determining the necessity and timing of neoadjuvant therapy. Additionally, these centers reported significantly fewer intraoperative complications (4.6% vs. 6.2%, *p* < 0.001), representing a relative reduction of 25.8% in complication rates. Hospital stays were also shorter in certified centers (19.46 ± 12.34 days vs. 20.24 ± 13.41 days, *p* < 0.001), suggesting more standardized surgical procedures and efficient postoperative care pathways. The reduction of nearly one day in the average hospital stay, while modest, may have significant implications for healthcare resource utilization and cost-effectiveness. Additionally, certified centers demonstrated significantly lower overall morbidity rates (37.5% vs. 40.4%, *p* = 0.002), representing a meaningful improvement in overall patient outcomes. Paradoxically, despite these advantages in preoperative and intraoperative care, several critical outcome measures showed no significant differences between certified and non-certified centers. General postoperative complications were comparable (18.8% vs. 20.2%, *p* = 0.068), as were specific complications related to rectal surgery (28.9% vs. 29.9%, *p* = 0.248). Particularly noteworthy was the similarity in anastomotic leakage rates (11.3% vs. 11.9%, *p* = 0.407) and reoperation rates (10.7% vs. 11.1%, *p* = 0.577), indicating equivalent surgical quality in these crucial aspects of rectal cancer care. Furthermore, our study found no significant differences in either short-term or long-term survival outcomes. The 30-day mortality rate was identical between groups at 2.6% (*p* = 0.869). More importantly, the 5-year overall survival rates showed remarkable similarity across all UICC stages: Stage I (89.1% vs. 88.3%, *p* = 0.800), Stage II (79.3% vs. 80.1%, *p* = 0.590), and Stage III (73.8% vs. 72.2%, *p* = 0.526). This pattern persisted even after adjusting for potential confounding factors through propensity score matching, suggesting that certification status alone does not significantly influence long-term survival outcomes.

Our findings regarding the quality of rectal cancer treatment in certified centers can be directly compared with several key studies. Bierbaum et al., analyzing a large cohort of over 160,000 colorectal cancer cases in Germany, demonstrated significantly lower mortality risk in certified centers (adjusted hazard ratio: 0.92; 95% CI: 0.89–0.95) [23]. Similar conclusions were presented by Völkel et al. in their study encompassing 47,440 patients [31]. In contrast to these studies, our results show no significant differences in 5-year overall survival between certified and non-certified centers (82.8% vs. 82.0%, *p* = 0.880). This discrepancy may be attributed to the fact that our study focused exclusively on rectal cancer, while previous studies analyzed colon and rectal cancer cases collectively. Andric et al., specifically examining the impact of certification on rectal cancer treatment in 563 patients, observed significant improvements in several areas post-certification, including the increased utilization of laparoscopic surgery (from 5% to 55%) and reduced preoperative hospital stay (4.71 vs. 4.13 days), with both parameters not reflecting quality of care. They also reported improved histopathological reporting and significant improvement in overall survival for UICC Stage IV patients [32]. Our findings corroborate some of these observations; we demonstrated better adherence to preoperative imaging guidelines and fewer intraoperative complications in certified centers. Similar to our findings, those of the WiZen study found no significant effect of certification on rectal cancer survival, although they reported improved 5-year overall survival for colon cancer patients [21]. Our observation of similar rates of postoperative complications and reoperations between certified and non-certified centers contrasts with some previous research, including a study of over 137,000 colorectal cancer patients in Germany, found lower 30-day mortality and reoperation rates in certified centers [33], suggesting that the benefits of certification may vary across different clinical settings and patient populations.

## 5. Conclusions

This study demonstrates that the certification of colorectal cancer centers has differential effects on various aspects of care. Certified centers showed improved adherence to preoperative imaging guidelines and reduced intraoperative complications. However, they did not demonstrate significant advantages in postoperative outcomes or long-term survival compared to non-certified hospitals.

These findings suggest that center certification effectively standardizes diagnostic procedures and reduces surgical complications, but its impact on postoperative care and long-term outcomes requires further optimization. Factors beyond certification status may play crucial roles in determining patient outcomes.

Future certification criteria might need to incorporate more specific measures targeting postoperative care quality and long-term survival. The continuous monitoring and adaptation of certification requirements could help identify and implement the most effective quality improvement measures. Additional research is warranted to elucidate the complex interplay of factors influencing cancer treatment outcomes and to guide the refinement of certification programs to maximize their benefit for patients.

## Figures and Tables

**Table 1:** Demographic and clinical characteristics of the study population.

Characteristic	Certified Centers (*n* = 3624)	Non-Certified Hospitals (*n* = 11,281)	*p*-Value
Age, years (mean ± SD ^1^)	68.17 ± 11.1	68.20 ± 10.69	0.966
Gender, *n* (%)			0.922
Male	2211 (61.2)	6905 (61.3)	
Female	1404 (38.8)	4368 (38.7)	
BMI ^2^, kg/m^2^ (mean ± SD)	26.63 ± 4.65	26.63 ± 4.42	0.706
≥1 preoperative risk factor, *n* (%)	2654 (74.5)	8870 (78.9)	<0.001
ASA ^3^ classification, *n* (%)			0.600
I	307 (8.6)	920 (8.2)	
II	1870 (52.3)	5909 (52.5)	
III	1317 (36.8)	4209 (37.4)	
IV	81 (2.3)	216 (1.9)	

^1^ standard deviation, ^2^ body mass index, ^3^ American Society of Anesthesiologists.

**Table 2:** Preoperative diagnostics and neoadjuvant therapy in certified and non-certified colorectal cancer centers.

Parameter	Certified Centers (*n* = 3624)	Non-Certified Hospitals (*n* = 11,281)	*p*-Value
Preoperative imaging, *n* (%)			
Endorectal ultrasound	2536 (70.7)	6560 (58.2)	<0.001
Abdominal CT ^1^	2995 (83.5)	9079 (80.6)	<0.001
Pelvic MRI ^2^	1399 (39.1)	3208 (28.5)	<0.001
Neoadjuvant therapy, *n* (%)	884 (24.4)	2719 (24.1)	0.956

^1^ Computed Tomography, ^2^ magnetic resonance imaging.

**Table 3:** Surgical quality indicators and tumor-related factors in certified and non-certified colorectal cancer centers.

Parameter	Certified Centers (*n* = 3624)	Non-Certified Hospitals (*n* = 11,281)	*p*-Value
Coning, *n* (%)			
Reported by surgeon	86 (2.6)	120 (2.3)	0.457
Reported by pathologist	129 (3.9)	193 (4.0)	0.878
M.E.R.C.U.R.Y. classification ^1^ (pathologist), *n* (%)			0.620
Grade 1	2802 (86.5)	4028 (86.5)	
Grade 2	354 (10.9)	523 (11.2)	
Grade 3	84 (2.6)	106 (2.3)	
pT stage, *n* (%)			0.019
pT1	585 (16.2)	1593 (14.2)	
pT2	1145 (31.8)	3590 (32.0)	
pT3	1729 (47.9)	5540 (49.3)	
pT4	149 (4.1)	510 (4.5)	
pN stage, *n* (%)			0.856
pN0	2350 (66.5)	7399 (66.5)	
pN1	770 (21.8)	2398 (21.5)	
pN2	413 (11.7)	1337 (12.0)	
UICC stage ^2^, *n* (%)			0.026
I	1455 (40.1)	4268 (37.8)	
II	985 (27.2)	3275 (29.0)	
III	1184 (32.7)	3738 (33.1)	

^1^ Magnetic Resonance Imaging and Rectal Cancer European Equivalence Study, ^2^ Union for International Cancer Control.

**Table 4:** Perioperative outcomes in certified and non-certified colorectal cancer centers.

Outcome	Certified Centers (*n* = 3624)	Non-Certified Hospitals (*n* = 11,281)	*p*-Value
Intraoperative complications, *n* (%)	166 (4.6)	694 (6.2)	<0.001
Postoperative complications, *n* (%)			
General complications	680 (18.8)	2276 (20.2)	0.068
Specific complications	1047 (28.9)	3376 (29.9)	0.248
Anastomotic leakage, *n* (%)	290 (11.3)	958 (11.9)	0.407
Reoperation, *n* (%)	381 (10.7)	1244 (11.1)	0.577
Length of hospital stay, days (mean ± SD ^1^)	19.46 ± 12.34	20.24 ± 13.41	<0.001

^1^ standard deviation.

**Table 5:** Short-term outcomes in certified and non-certified colorectal cancer centers.

Outcome	Certified Centers (*n* = 3624)	Non-Certified Hospitals (*n* = 11,281)	*p*-Value
30-day mortality, *n* (%)	95 (2.6)	290 (2.6)	0.869
Overall morbidity, *n* (%)	1359 (37.5)	4554 (40.4)	0.002

**Table 6:** Long-term outcomes in certified and non-certified colorectal cancer centers.

Outcome	Certified Centers (*n* = 3624)	Non-Certified Hospitals (*n* = 11,281)	*p*-Value
5-year overall survival, % (95% CI)			
All stages	82.8 (80.1–85.5)	82.0 (79.7–84.3)	0.880
UICC Stage I	89.1 (85.8–92.4)	88.3 (85.4–91.2)	0.800
UICC Stage II	79.3 (74.0–84.6)	80.1 (75.8–84.4)	0.590
UICC Stage III	73.8 (66.5–81.1)	72.2 (66.3–78.1)	0.526

**Table 7:** Long-term outcomes before and after certification.

Outcome	After Certification	Before Certification	*p*-Value
5-year overall survival, % (95% CI)			
All stages	80.1 (76.0–84.2)	84.4 (81.1–87.7)	0.198
UICC Stage I	88.3 (83.2–93.4)	90.9 (86.6–95.2)	0.458
UICC Stage II	76.9 (68.8–85.0)	85.5 (79.4–91.6)	0.088
UICC Stage III	71.9 (64.0–79.8)	74.5 (67.0–82.0)	0.832

**Table 8:** Comparison of outcomes before and after certification.

Outcome	After Certification	Before Certification	*p*-Value
Preoperative imaging, *n* (%)			
Endorectal ultrasound	447 (67.2)	338 (59.2)	0.003
Abdominal CT	558 (84.2)	467 (81.9)	0.297
Pelvic MRI	280 (42.1)	172 (30.1)	<0.001
Neoadjuvant therapy, *n* (%)	234 (35.3)	190 (33.3)	0.913
Intraoperative complications, *n* (%)	23 (3.5)	26 (4.6)	0.331
Postoperative complications, *n* (%)			
General complications	126 (18.9)	94 (16.5)	0.255
Specific complications	179 (26.9)	175 (30.6)	0.148
Anastomotic leakage, *n* (%)	54 (11.6)	52 (12.7)	0.638
Reoperation, *n* (%)	69 (10.4)	57 (10.0)	0.813
Length of hospital stay, days (mean ± SD)	19.7 ± 13.82	19.7 ± 12.42	0.432
Overall morbidity, *n* (%)	241 (36.2)	218 (38.2)	0.482
30-day mortality, *n* (%)	20 (3.0)	18 (3.2)	0.883

**Table 9:** Multinomial regression analysis of UICC stage distribution (*n* = 14,905).

Predicator	UICC Stage II vs. I		UICC Stage III vs. I	
	Adjusted OR	*p*-Value	Adjusted OR	*p*-Value
Certified Status				
Certified (vs. Non-Certified) Patient Characteristics	0.86 (0.78–0.95)	0.026	0.92 (0.84–1.01)	0.526
Age (years) ^1^	1.02 (1.01–1.03)	<0.001	1.01 (1.00–1.02)	0.042
Male gender	1.18 (1.08–1.29)	<0.001	1.22 (1.12–1.33)	<0.001
BMI (kg/m^2^) ^1^	1.01 (1.00–1.02)	0.084	1.00 (0.99–1.01)	0.706
Clinical Factors				
ASA Classification (vs. I)				
-II	1.15 (1.01–1.31)	0.038	1.19 (1.04–1.36)	0.012
-III	1.28 (1.11–1.47)	0.001	1.35 (1.17–1.56)	<0.001
-IV	1.42 (1.12–1.80)	0.004	1.58 (1.24–2.01)	<0.001
≥1 Preoperative Risk Factor	1.26 (1.15–1.38)	<0.001	1.31 (1.19–1.44)	<0.001

^1^ Continuous variables.

## Data Availability

The data presented in this study are available in this article.
